# Placing assistive technology and telecare in everyday practices of people with dementia and their caregivers: findings from an embedded ethnography of a national dementia trial

**DOI:** 10.1186/s12877-020-01896-y

**Published:** 2021-02-15

**Authors:** Matthew Lariviere, Fiona Poland, John Woolham, Stanton Newman, Chris Fox

**Affiliations:** 1 Centre for Research on Health and Social Care, School for Policy Studies, University of Bristol, Bristol, BS8 1TZ UK; 2 School of Health Sciences, University of East Anglia, Queen’s Building, Norwich, NR4 7TJ UK; 3 Health and Social Care Workforce Research Unit, King’s College London, Kingsway, London, WC2R 2LS UK; 4 School of Health Sciences, City University of London, Northampton Square, London, EC1V 0HB UK; 5 Norwich Medical School, University of East Anglia, Norwich, NR4 7TJ UK

**Keywords:** Qualitative methods, Implementation, Uptake, Home, Care

## Abstract

**Background:**

Policy makers and care providers see assistive technology and telecare as potential products to support people with dementia to live independently in their homes and communities. Previous research rarely examined how people with dementia and their caregivers actually use such technology. The study examined how and why people living with dementia and their caregivers used assistive technology and telecare in their own homes.

**Methods:**

This study used an ethnographic design embedded within the NIHR-funded Assistive Technology and Telecare to maintain Independent Living At home for people with dementia (ATTILA) randomised controlled trial. We collected 208 h of observational data on situated practices of ten people with dementia and their ten caregivers. We used this data to construct extended cases to explain how technologies supported people with dementia in home and community settings.

**Results:**

We identified three themes: placing technology in care, which illustrates how people with dementia and caregivers ‘fit’ technology into their homes and routines; replacing care with technology, which shows how caregivers replaced normal care practices with ones mediated through technologies; and technology displacing care and everyday life, which highlights how technologies disrupted the everyday lives of people with dementia.

**Discussion:**

This study exemplifies unintended and unanticipated consequences for assistive technology and telecare uptake in ‘real world’ community-based dementia care. It underlines the need to identify and map the context of technological provision over time within the changing lives of people with dementia and their caregivers.

## Background

Assistive technology and telecare (ATT) are championed as possible interventions to support people living with dementia to live independently and safely within their homes and wider communities [1]. Assistive technology refers to ‘any device or system that allows individuals to perform tasks they would otherwise be unable to do or increase the ease and safety with which tasks can be performed’ [2]. However, relatively little research has examined how, and why, people with dementia and their caregivers actually use these technologies in their everyday lives and how such experiences may affect their wellbeing and ability to sustain their community-based care arrangements. This article describes findings and implications of an ethnographic study embedded within a national dementia trial examining how people with dementia and caregivers actually used technology in their everyday lives.

In England, *The Five Year Forward View* outlined the strategy for the health services in England suggesting particular areas where innovation may lower costs while increasing care quality [3]. This strategy highlighted the need for NHS England to exploit novel technologies within future care arrangements to attain these outcomes. The current evidence base for some technology, like telecare, does not confirm their appropriateness or efficacy.

The Whole System Demonstrator trial was one such trial investigating the effectiveness of telehealth and telecare products for people with chronic illnesses, like diabetes, with a notable exclusion of people with dementia. For telecare, the trial presented mixed results. Telecare was more expensive than usual care [4]. Telecare also did not reduce: rates of participants’ hospital use, length of inpatient hospital stay, or admissions into institutional care settings [5]. However, telecare reduced the decline in trial participant’s mental health related quality of life over the twelve-month study period [6]. Despite these results, primary care and social services continue to deploy telecare as interventions for mitigating unmet health and social care needs [7]. However, recent research has identified widespread inaccuracies reporting Whole System Demonstrator findings as a contributing factor to continuous implementation of telecare and other technology in health and care [8].

Within the context of community-based care for older people living with a dementia, there is a small, but growing, body of literature exploring how technology may support them and their families. Reviews have identified categories of technology which *may* support functional capabilities for people with dementia (e.g. memo minders, information and communication technologies) or help caregivers organise and coordinate their caring responsibilities (e.g. care coordination apps, messenger services) [9, 10]. However, the range of evidence for each technological category varies widely with relatively few technologies evaluated through randomised controlled trials or systematic reviews (Moyle, 2019) [11].

Previous studies have highlighted how assistive technologies may improve functional capabilities, including cognition, for people living with dementia to carry out specific ‘tasks’ or ‘activities of daily living’ related to personal hygiene, eating, ambulation and other everyday facets of life [12–18]. However, this previous research did not specify people with dementia as participant groups in their respective studies. Instead, they included people with other progressive or acquired conditions limiting cognitive functionality.

One interview study with occupational therapists and caregivers explored potential types of assistive technologies to help people with dementia engage in personally meaningful daily activities [19]. The study identified enablers for assistive technology in dementia care as perceived effectiveness, relative low cost of the technology, and familiarity with the technology. Conversely, the study identified barriers for assistive technology in dementia care related to caregiver burden, poor installation and fit within domestic environments, and a perceived lack of safety. While this study accounts for how health professionals and family caregiver perceive the role of assistive technology to support people with dementia, its exclusion of people with dementia from the study raises questions about how people with dementia viewed the utility of such technology and imbued its meaning in their everyday activities. Caregivers may have limited insight into what people with dementia want, and their reasons for using assistive technologies. Direct involvement of people with a dementia in research would highlight how and why they chose to use assistive technology, uncovering details about processes of sense-making entwined within the context of their lived experience with the illness.

A study with oven timers identified how technology deployed to support independence and personal safety may create additional, unforeseen issues when placed within everyday practices of people with a dementia [20]. The study found people with dementia rarely decided whether, or how, to install oven timers despite the direct impact these decisions would have on their own kitchen habits. People with dementia experienced unforeseen difficulties using the oven timer, including, misunderstanding its audible and visual alarms or the intended use of its ‘magnetic key’ to reset the oven.

Other research has explored the expressed concerns of people with dementia about how technology affects their everyday lives. One qualitative study with people with dementia, caregivers, and care professionals identified ethical problems arising from how people with dementia use assistive technologies [21]. People with dementia voiced dislike for what they perceived as unnecessary forms of surveillance through caregivers’ use of assistive technology. In contrast, caregivers argued assistive technology could potentially mitigate risks thereby providing a reasonable justification for intruding in the lives of people living with a dementia. These findings illustrate the importance of including both caregivers and people with dementia in research to explain dissonances in narratives and practices with technology, tensions between balancing care needs and priorities in an individual’s everyday life.

Recent research has identified additional layers of complexity with the delivery and uptake of technology in dementia care by people with dementia and family caregivers [22–24]. People with dementia, family caregivers and general practitioners may experience difficulty acquiring information about available assistive technology [24]. Individual localities may have assistive technology and telecare provided by health services, social care providers, or a hybrid service model involving both provider organisations with regional variation of assistive technology products and different price points [22]. The lack of familiarity with organizations and contacts relevant to acquiring assistive technology may inhibit the capacity for people with dementia to acquire assistive technology and receive suitable instruction about their after they are in place. When people with dementia received assistive technology, they often attempted to ‘fit’ these into their lives rather than working with the ‘off the shelf’ product to construct ‘DIY assistive technologies’. [23] Such work draws on the concept of ‘bricolage’, the process of assembling tools with readily available and ready-to-hand materials, first described by anthropologist, Claude Levi Strauss, and applied more recently in digital health research [25, 26]. Previous studies have often relied on cross-sectional interview methodologies paying limited attention to the practices and decisions informing how people with dementia and their caregivers use assistive technology over time as their care and support needs change. The study presented here, A Collaborative COMMunity-based ethnography Of people with Dementia and their caregivers using Assistive technology and Telecare in England (ACCOMMODATE), aimed to address this empirical research gap drawing on a sub-sample of caregivers and people with dementia participating in a national randomised controlled trial evaluating the efficacy of ATT for people living with dementia and their caregivers.

‘Practice’ is a key concept in the social sciences to explain the relationships between human action and social structures. Pierre Bourdieu, a prominent practice theorist, claimed people could act contrarily to the social structures and systems which ordered their reality thereby expressing human agency [27, 28]. In his theories of practice, Bourdieu often explored what he called ‘the habitus’, the assemblage of social structures and intentional acts of individuals exercising their agency [27]. According to Bourdieu, habitus framed how society provided humans with certain ‘dispositions’. These dispositions, in turn, structured how humans could act in specific situations – within a range of spatial, temporal or social dimensions. However, dispositions were not fully fixed or inert; human intent and actions could change them [28]. Such theories continue to dominate one the central tensions in the social sciences: the relationship between social structure and agency.

Other scholars defined ‘practices’ more broadly as ‘anything people do’ with ‘unintentional or intentional political implications’ which includes virtually all human activities [29]. More recent social theorists argued for concepts of ‘practice’ as ‘embodied, materially mediated arrays of human activity centrally organised around shared practical understanding’. [30]

For this study, the team drew on this latter conceptualisation of ‘practices as arrays of human activity’ to examine the role of assistive technology and telecare in community-based dementia care. This formulation of practice highlights the material facets of ‘doing’ through its focus on observations combined with uncovering processes of sense-making tied to ‘practical understanding’.

### Aims and research questions

The study aimed to exemplify and examine how and why people with dementia and their caregivers used or chose not to use ATT in their lives and ways ATT use affected their environments and relationships.

To address this research aim, the study team sought to answer three research questions:

1. How and why do people with dementia and their caregivers use assistive technology and telecare (ATT) at home?
2. How does ATT ‘fit’ into peoples’ lives and care arrangements in their homes?
3. How do ATT technologies affect peoples’ lives and care in their homes?

## Methods

This study used a qualitative focused ethnographic observational, longitudinal design to investigate how and why people with dementia in the intervention arm of the ATTILA trial used or did not use the ATT offered to them as well as changes to how they used ATT over time.

### Study design

Ethnographic approaches have commonly relied on sustained fieldwork, where a researcher takes part in a group’s practices while observing them, so as to rigorously interpret how people make sense of their everyday lives and social systems [31]. The ethnographic approach used in this study drew on recent multidisciplinary research in trials to design a study ‘embedded’ within a broader based research programme to collect focused observational data on situated practices of people with dementia and their caregivers when using ATT in their everyday lives [32–41]. We used this data to construct in-depth extended cases of how people with dementia and caregivers used (or chose not to use) these technologies, to help explain how and how far specific technologies for supporting people with dementia may be relevant in the context of their everyday situation and interactions in home and community settings.

To provide trustworthy findings and interpretations, we formulated credible, transferable, dependable, and confirmable processes for data collection and analysis through:

- Extensive and intensive data collection with participants (detailed below) and critically-discussed anonymised data (credibility) findings within the study teams (credibility, dependability).
- Noting contextualised detail participants’ situated practices and how these may affect interpretation of findings to other contexts (transferability).
- Reflecting on the researcher’s role in the process and how this could affect interpretations. Detailed fieldnotes and accounts of the research context to allow future research to confirm, challenge or, otherwise, build on findings from this study (confirmability) [42, 43].

Ethics approval for the study was granted by the National Research Ethics Services Committee East of England, Norfolk (15/EE/0015) on 3rd February 2015.

### Sample

A purposive sampling strategy was used to select potential participants from the wider ATTILA study population able to provide data relevant to examine care practices and specific reasons for the extent and ways of their uptake of diverse technological interventions.

This strategy, therefore, incorporated the ATTILA inclusion and exclusion criteria (Table 1) but also ethnographic-specific purposive sampling criteria which would provide contextually relevant and diverse types of participants’ experiences from three characteristics:

- Severity of the person’s dementia, as recorded by ATTILA research workers;
- Type of family relationship between the caregiver and person with dementia; and
- Types of assistive technology and telecare equipment provided to the person with dementia.

**Table 1:** ATTILA Inclusion and Exclusion Criteria

**Inclusion criteria**	• Clinical dementia rating of 1, 2 or 3 • Fair access to care services (FACS) assessment indicates significant need • Working telephone line connected to the home
**Exclusion criteria**	• Person already receiving an ATT intervention or has previously been provided with ATT but failed to use it. • Person has an unstable medical condition.

### Recruitment

The ethnographic team recruited potential participants alongside three ATTILA researchers working in three distinct local authorities in east and southeast England, consisting of urban and rural populations within areas of relative wealth and deprivation [44]. These are pseudonymised to ensure anonymity and confidentiality into ‘Shire’, ‘Metropolitan’ and ‘Coast’. ‘Shire’, ACCOMMODATE participants from this area lived in large villages or small market towns with independent vendors and occasional high street shops. ‘Metropolitan’, a major city divided into different districts. Study participants lived in two adjoining districts with historical poverty but more recently subject to gentrification which is transforming this area. ‘Coast’, two counties with a seaside border where ACCOMMODATE participants lived predominately in large market towns or in villages near major regional city hubs.

These brief settings descriptions highlighted distinct features of the wider areas where people with dementia lived, to contextualise their wider relations with their environment.

The fieldworker (ML) collaborated with an ATTILA research worker to identify prospective ACCOMMODATE participants from the existing ATTILA sample. The fieldworker selected people based on how they aligned with the purposive sampling criteria for ACCOMMODATE. Initially, the fieldworker selected the next available ATTILA participant to coincide with the pre-arranged follow-up visit for the trial’s local research worker. However, the fieldworker more actively selected later cases to ensure maximum variation across all three purposive sampling criteria. For example, the fieldwork selected participants who received different ATT products; participants with more advanced dementia, or participants with different social relationships (e.g. spouse, parent-child) living in the same or separate homes to each other. The fieldworker then attended the pre-arranged ATTILA follow-up visit with the area’s local research worker to meet with prospective participants to discuss participation in the ethnographic sub-study. He sought additional informed consent from people with dementia and their caregiver to take part in the ethnography sub-study or a consultee declaration from the caregiver for the person with dementia to participate in this embedded sub-study [45]. The fieldworker continually renegotiated informed consent during each subsequent monthly visit of his independent fieldwork. The fieldworker assessed the mental capacity of each person with dementia to understand information about the study, weigh the benefits and potential risks involved with their participation to decide whether they wished to take part. Such processes adhered to the Mental Capacity Act Code of Practice [46].

### Participant-observation

The study included nine ethnographic cases [47]. Each consisted of at least one person with dementia (*n* = 10) and their caregiver (*n* = 10). Two cases included more than one caregiver (the Campbells included two caregivers) or more than one person with dementia (the Stewarts included two people with dementia). Data collection involved up to six, monthly visits to the home of each person with dementia. These monthly visits lasted between one and 5 h. A total of 208 h of observations took place over 60 visits. Each visit involved the fieldworker observing routine, everyday practices of people with dementia and their caregivers. Although the fieldwork was particularly interested in how, and to what extent, either the person with dementia and/or their caregiver used ATT, he sought to understand how participants used them within the context of everyday life.

The researcher’s conversations with participants also formed an integral part of participant-observation fieldwork. These conversations were unstructured and ad hoc with participants to elicit their reasons (i.e. sense-making) for using or not using ATT or additional information about their routine. These conversations provided contextual details about how people with dementia and caregivers made sense of their activities as they occurred. Instead of asking them ‘what activities they would normally take part in each day?’, the researcher could seek more information as the activity took place, such as, “What are you doing?” Engaging with people with dementia in this way, meant the researcher could seek situated and specific information about routine, everyday practices as well as extraordinary events in participants’ lives. With this methodological approach, people with dementia did not have to rely on retrospective reflections about their everyday lives. They could respond to questions focused on the immediacy of the situation they participated in at any single given moment.

The fieldworker wrote initial notes from these observations and conversations in a field journal during or immediately after the visit with rough maps drawn to illustrate objects and peoples’ places in domestic settings [48, 49]. These notes, or ‘jottings’, served as an *aide-memoire*, which supported by reflective reviewing practices, were used to construct fieldnotes as ‘thick descriptions’ [50]. These fieldnotes represented what the fieldworker observed and did during his time with each person with dementia and their caregiver. ‘Thickness’, here, arose from attention to detail and context of participants’ everyday practices.

### Analysis

The study team analysed each case using situational analysis of longitudinally extended cases [51, 52]. Analysis was assisted through computer-assisted qualitative data analysis software, Nvivo, to help identify and collate themes using visual (maps) and textual data (fieldnotes). The main themes identified from memos highlighted how people with dementia and caregivers attempted to fit ATT into their everyday practices leading to placing, replacing and displacing care highlighted by how practices with ATT appeared to alter how participants interacted with each other and used spaces inside and outside the home. Focused coding was used to identify instances of these themes within each ethnographic case, which informed comparisons within and across cases to contextualise specific instances of these analytical themes. The findings are presented here as condensed cases with excerpts from field notes to highlight analytical features seen as common to several cases. These are depicted further through indicative maps.

## Results

Table 2 provides descriptions of the nine ethnographic cases in terms of their location, the severity of the person’s dementia, nature of participant’s family or care relationship, and types of assistive technologies and/or telecare products in place. Table 2 also specifies the total amount of time spent with each case group. Some cases had demonstrably fewer hours of observation for varying reasons, such as hospitalisation of a participant (The Drapers), death of a participant (Violet and Rose), or limited time available to the caregiver due to extensive travel required to visit the home of the person with dementia (The Smiths). Ethnographic cases were evenly distributed across different levels of dementia severity (i.e., 3 mild, 3 moderate and 3 severe cases). They most commonly received a falls detector (*n* = 6) and door sensors (*n* = 4). Key safe (*n* = 3) and calendar-clocks (*n* = 3) were also less frequently provided. All participant names are pseudonyms to ensure confidentiality and anonymity.

**Table 2:** Description of Ethnographic Cases (Adapted from Lariviere, 2018)

Case names and location	Dementia severity	Relationship	Assistive technology and telecare
Clydes – Coast (33 h of observation)	Moderate	Father (person with dementia) lived alone in his own house. Son and daughter-in-law (caregivers) lived in separate house, but visited most days.	Automatic falls detector (wristband model), keysafe
Drapers – Coast (14 h of observation)	Mild	Mother (person with dementia) lived alone in her own home. Son (caregiver) lived in his own separate home but visited her for up to 6 hours every day.	Calendar-clock, bed sensors automatic falls detector, falls alarm (wrist version; replaced pendant after first visit), keysafe
Stewarts – Coast (28 h of observation)	Moderate (both individuals)	Mother and father (people with dementia) lived in extension of the daughter’s house (caregiver).	Door sensors
Betty and Rose – Shire (10 h of observation)	Severe	Betty (caregiver) is Rose’s (person with dementia) neighbour. They each lived in their own house.	Automatic falls detector (pendant), keysafe
Anthony and Mrs. Archer – Metropolitan (28 h of observation)	Severe	Mrs Archer (person with dementia) lived in a sheltered housing flat. Anthony (caregiver; friend of family) lived his own flat in the same neighbourhood as Mrs. Archer. He visited her a few days per week.	GPS tracking device, calendar-clock, automatic falls sensor (pendant), cooker-timer
Campbells – Metropolitan (30 h of observation)	Severe	Son (caregiver) lives in mother’s (person with dementia) home	Bed sensor, door sensor/alarm, pendant alarm
Browns –Shire (29 h of observation)	Mild/MCI	Wife (caregiver) shares house with husband (person with dementia); daughter (caregiver) and son-in-law live in annexe	Door sensors, object finder
Anansis – Metropolitan (22 h of observation)	Moderate	Father (person with dementia) lives alone in a flat; daughter (caregiver) visits him regularly from her home across the city.	Automatic falls detector (pendant), GPS ‘watch’ and pendant (Buddi)
Smiths – Shire (14 h of observation)	Mild	Father lives alone in his own house. Daughter (caregiver) lives with her family in village from another county.	Wrist alarm, Automatic falls detector (waist), calendar-clock (self-purchased), door sensors, networked smoke alarm, activity monitoring sensors and software (JustChecking)

Three key themes were identified as relevant to understand the ATT-relevant and care-relevant everyday practices, routines and relationships of study participants:

1. Placing technology in care
2. Replacing care with technology
3. Technology displacing care and everyday life

### Placing technology in care

The theme, ‘placing technology in care’, represents instances where people with dementia and/or caregivers fit ATT products into their existing care arrangements. It addresses participants’ processes and practices in incorporating and adapting technologies into their everyday lives with varying degrees of success.

**Fig. 1:**
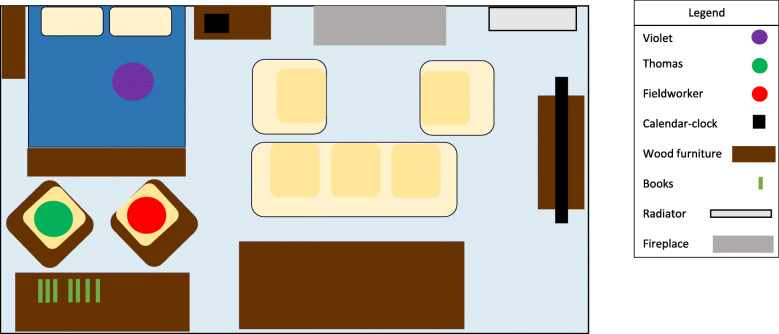
This figure illustrates the placement of the fieldworker (Matthew) in the sitting room of Violet Draper, a person living with dementia in Coast, during his first fieldwork visit to her house. Thomas Draper, her middle-aged son and primary caregiver, is also present. This figure typifies the placement of all three actors during the first two fieldwork visits completed by the study team. Matthew and Thomas sitting on two adjacent chairs near the bed of Violet. Thomas moved Violet’s bed prior to the study’s fieldwork so Violet did not have to ascend/descend the stairs to access her bedroom following a hip replacement operation. In the figure, one observable assistive technology product was an electronic calendar-clock, which displayed the date and time to help orientate Violet. The spatial elements of people and objects in this figure highlights how caregivers and people with dementia adapt spaces and technology to ‘fit’ into placements of care

The Drapers’ case, showed initial troubles in how they placed a falls detector within their pre-existing everyday practices. Prior to the fieldworker commencing data collection, the person with dementia, Violet Draper, had received a falls detector pendant, after experiencing several falls. Around the time research visits began nearly 12 weeks after the local authority provided the pendant alarm, she had another fall, but the alarm did not trigger. Her son and caregiver, Thomas, told the fieldworker during this first visit that Violet had decided not to trigger the alarm manually as she “did not want to be a bother” to him or the emergency response services. Thomas also commented that his mother frequently forgot to wear the pendant or took it off in the evening after he moved her bed down to the sitting room (see map in Fig. 1). After this fall, Thomas changed two elements of his mother’s care. First, he asked the local ATT provider to swap the pendant-style detector for one worn around the wrist. Second, he reminded Violet every day to “press the button” if she ever fell again. When the fieldworker witnessed Thomas reminding Violet to “press the button”, she often nodded along in agreement.

Next time the fieldworker visited in late autumn, things appeared more settled. Thomas showed him around Violet’s home giving a tour of the ground floor of the detached house in a quiet village. They spent the majority of the visit in the lounge-bedroom where Violet sat on a bed often reading the newspaper. Thomas and the fieldworker often exchanged stories about life in the village and the surrounding area punctuated by Thomas getting up to make cups of tea for Violet. During this visit, the fieldworker again observed Thomas reminding Violet to “press the button” of her falls detector if she ever falls again. Violet grumbled but gradually nodded in agreement.

A couple of months later just before Christmas, the fieldwork visited Violet’s home again. However, Violet was not available, only Thomas was there. Thomas told the fieldworker that Violet had a “really bad fall” in which she broke her leg. He recounted how the falls detector did not detect the fall automatically, but Violet “remembered to press the button” to activate the falls detector which contact first responders and Thomas.

This case highlights how both Thomas and Violet acted to make the falls detector fit into their lives. Their case illustrates work of caregivers to instruct and to reinforce instructions to ensure ‘successful’ implementation of ATT that may otherwise have been invisible to care workers. Such reinforcement of instructions was even more important in this instance as the falls detector did not activate automatically. This case demonstrates the persisting importance of social connections and support for enacting ‘technology-enabled’ care systems.

This raised the issue of the selection of appropriate technologies to enhance the safety of people with dementia. A key distinction here is between ‘passive’ devices that automatically trigger, not requiring the user to perform any actions, and devices that require an action to be performed to activate them. It seemed important to consider the appropriateness of passive devices for people with dementia, especially where they are either reluctant to trigger alarms or have memory difficulties which leading them to fail to remember to trigger a device. It also highlighted issues about the reliability of technology. With user-activated technology, it is crucial that people with dementia and caregivers can trust technology to function appropriately when they use it as intended. If technology does not function, then the consequences for such failure can lead to unidentified falls or other crises, and reduced trust in other alternative, perhaps more suitable, interventions. For understanding implementation and uptake, unreliable technologies can lead people to reject or abandon its use.

By contrast, the Stewarts’ case illustrated how people with dementia or caregivers may appropriately place assistive technology, yet find that other objects in the home may be more suitable for addressing problems when they arise. Mary and Michael were a married couple where both had dementia. Their daughter, Sally, moved them into an annexe of her home to support them full-time. During one fieldwork visit, Sally asked her parents for the date. Neither Michael nor Mary knew the date. They also did not appear to notice the nearby calendar-clock that had been provided to display this information but did perceive wooden calendar blocks across the room, which helped orientate them. Michael told Sally the correct date.

This case highlighted the importance of using established material to achieve the appropriate outcome, in this case, orientation to time. The calendar-clock did not disrupt or disorientate Michael and Mary, but neither did this technology actively facilitate them to be orientated to the current date and time. Although someone still had to interact with the regular wooden calendar to change the date each day, its familiar location and design may have more easily supported their orientation, because it relied in part on older memory and was perhaps more readily recognisable than more recent digital counterparts.

The Browns’ case raised further questions about how researcher and practitioners come to define ‘use’ of ATT. Sam Brown, a person with mild cognitive impairment (MCI), had a memo minder in his house entrance. A recording of his daughter’s voice reminded him to lock the front door whenever anyone walked in front of the infrared motion sensor. Sam told the researcher that he always remembered to lock the door because of it. Sam also shared his home with his wife and adult daughter and son-in-law, who lived in a converted garage annexe. The other household residents became annoyed with the memo minder repeatedly going off whenever they went to put on or remove their shoes and outerwear. Sam decided to turn off the recording, but leave the memo minder in its place next to the front door. He insisted, and the researcher observed and confirmed, multiple times, that seeing the now-silent memo minder still beside the door, reminded him to close and lock it when he left the house to go back home.

Such practices blurred the ‘use’ of ATT and its ‘non-use’. They demonstrated how devices may be adapted to local circumstances suitable to the person with dementia’s level of cognitive functioning. Although the person with dementia switched off this device, its co-location with him in its ‘appropriate’ place provided the prompt he needed to remember to lock up. This case illustrated how people are able to actively accommodate technology to work within their shared spaces and their relationships with others, including their caregivers.

### Technology replacing care

This theme, ‘technology replacing care’, addressed how caregivers, through engaging with ATT, replaced or reconfigured their practices of caring for people with dementia.

Arthur Clyde, an older person with dementia, received a falls detector from his local authority. His son and daughter-in-law, Mark and Cathy, visited his home every weekday to work from the front room of his house that they converted into an office for Mark’s business. Mark also used to visit his father at least 1 day over the weekend to see whether he remembered to heat up and eat his pre-prepared meals. However, after Arthur started to wear the falls detector around his wrist, Mark visited his father less frequently. Mark told the researcher that he had ‘peace of mind’ that the call centre would notify him if his father had a fall. Mark decided instead to phone Arthur on Saturdays and Sundays, to ask him whether he ate his meals instead of visiting to confirm this.

This case illustrated how caregivers may change their care practices for a person with dementia after they introduce ATT into their arrangements. Here the caregiver visited his father less frequently and relied on the falls detector and telephone to monitor his father with dementia. Monitoring practices changed and were mediated through technologies rather than face-to-face interactions. Caregivers’ sense of security, often articulated as their ‘peace of mind’, was a common response across cases and one of the intended benefits of introducing ATT devices in dementia as is also illustrated in the following case.

**Fig. 2:**
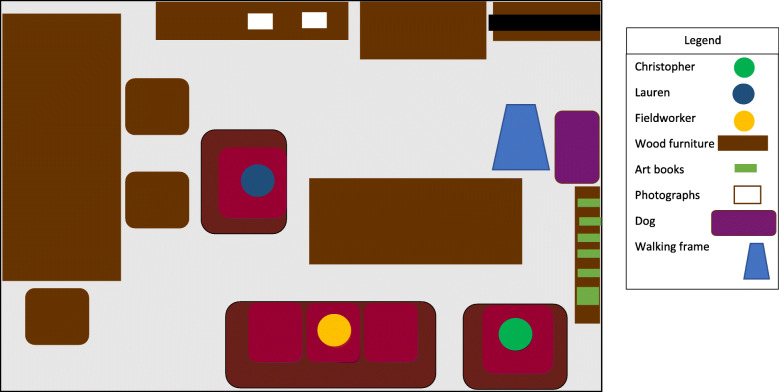
This figure illustrates the placement of the fieldworker (Matthew) in the sitting room of Christopher Smith, a person living with dementia in Shire, during the fifth visit to his house for the study. Lauren Smith, his middle-aged daughter and primary caregiver, is also present. This figure typifies the placement of all three actors during the previous fieldwork visits completed by the study team. Matthew often sat on the couch with Lauren sitting on the chair in front of him while Christopher sat on his favourite chair in the corner of the room. In previous visits, the activity monitoring system had a sensor placed on the wall between the two photographs on the chest of drawers opposite the couch. During this visit, the fieldworker observed Christopher’s dog frequently jumping on the couch beside him which placed it directly in front of the motion sensor. As Matthew never observed Christopher leaving his chair during his subsequent visits, he asked Lauren about whether the sensor could be picking up the dog’s movements instead of her father. Such observations highlight how when we use remote monitoring technologies, people cannot always ensure the quality and accuracy about what they imagine the observe (a person with dementia) with potential realities (a dog on the couch). This could jeopardise the security and peace of mind ATT offers caregivers and other user groups

In the Smiths’ case, Lauren had the local service provider install an activity monitoring system in the living room of her father’s bungalow. Lauren thought her father, Christopher Smith, frequently got up from his favourite chair to walk around the house based on activity reported on the monitoring system’s accompanying app for her tablet. Lauren told the researcher that she had “peace of mind” that her father remained active even when home alone, especially as she lived in another county distant from her father. During the researchers visits Christopher was rarely seen to move from his chair (see Fig. 2 map). During the penultimate visit (month 5), Lauren and the researcher noticed the dog jumping on the couch. The fieldworker asked Lauren whether her App ‘picked up’, i.e. registered monitor activity, even though no-one had moved except for the dog. The fieldworker asked Lauren whether she possibly monitored the dog instead of her father which led Lauren to wonder aloud how frequently her father really left his chair.

The Smiths’ case highlights how people may use these devices for reassurance and peace of mind. In this case the imprecisely-placed product led to inaccurate information and misguided reassurance. Once this was established, the caregiver’s ‘peace of mind’ became replaced by concern as she could no longer be certain whether the activity monitoring system monitored only her father’s movement or also those of other people or animals. In contrast to the other two cases exemplifying this theme, the case of the Campbells demonstrates how people can independently adopt other technologies and how this will also shape their care practices. Kenneth Campbell shared his home with, Lillian his mother with dementia. They were offered door and bed sensors, but Lillian tore out the cable from the bed sensor from under the mattress. Kenneth independently purchased and used a CCTV system to monitor the downstairs rooms of the house, where his mother lived, through monitors in his living room upstairs.

The Campbells’ case shows how caregivers may provide care with the addition of technologies. Notably here, the ATT did not appear to fit into the lives of Kenneth or Lillian it may have acted as a prompt and led to Kenneth adapting security equipment, i.e. CCTV, as a means to monitor his mother in their home. This case raises further questions about how we characterise means to monitor people with dementia in their home as appropriate yet still ensure dignity and safeguard them against harm. It also calls into question whether caregivers’ work here may have changed rather than diminished.

### Technology displacing care and everyday life

The final theme, ‘technology displacing care and everyday life’, represents cases where people with dementia experienced their care arrangements and everyday practices as being displaced from their usual routines by ATT.

In the Anansi case, technology seemed to constrain how William Anansi could engage as he wanted with his wider community. William received a GPS tracking system from his local council. Claire, his daughter and primary caregiver, told the researcher that she hoped this device would allow both her father to leave the house when he wished but also for her to locate him if he became lost. During one research visit, William left his flat without telling Claire. She called the call centre for the GPS tracking device, which located him in a nearby market where he frequented for his favourite Caribbean cuisine. Claire called her father on his mobile to tell him to return home. She also told the call centre operator to contact him through the speaker on the GPS tracking device. William initially did not answer any calls. After 10 min of her calling him, he answered his mobile and told Claire that he had had lunch. Claire again told him to return home. The call centre operator confirmed that William appeared to be on a bus on his way back to his flat.

This case illustrates how caregivers can use technologies to ensure safety but also can affect how people with dementia interact with spaces outside their home and engage with their wider community. This case illustrates how people can attempt to control movements and behaviour of people with dementia. New challenges and concerns for caregivers may be raised rather than be removed by ATT in order to enable the individual with dementia to access their wider community in a safe and secure way.

**Fig. 3:**
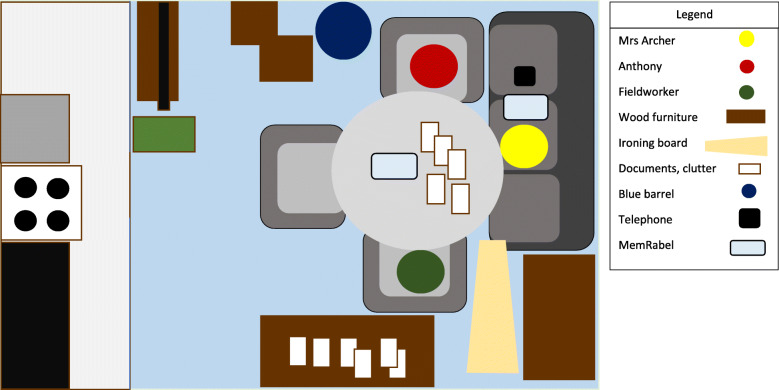
This figure illustrates the placement of the fieldworker (Matthew) in the sitting room of Mrs. Archer, a person living with dementia in Metropolitan, during the fifth visit to her sheltered living flat for the study. Anthony, a friend of the family from church and primary caregiver, is also present. This figure typifies the placement of all three actors during the first two fieldwork visits completed by the study team. Matthew and Anthony often sat around the small round dining table while Mrs. Archer sat on the couch adjacent to them. During this fifth visit, Anthony showed Matthew the inside of the kitchen where a fire had taken place the previous evening before the fieldworker’s visit. There was a small crater burned into the worktop between the oven and sink. Sooty, smoke damage extended up the walls and covered all of the overhead cabinets. Anthony explained to Matthew the story Mrs. Archer had told him: Mrs. Archer’s granddaughter stayed over the previous night and when she felt hungry she started to fry chicken, however, she fell back asleep only to awaken to smoke and the fire. It appeared the hot pan was placed on the worktop. Anthony speculated that this could have been avoided if the pot was left on the stovetop as the automatic cooker shut off device would have turned it off. Due to concerns for Mrs. Archer’s and safety of the facility’s other residents, the site manager decided to remove the oven with Anthony’s approval

The final case, Anthony and Mrs. Archer, represents another example of how technology displaces care and everyday life. Mrs. Archer, an older woman with dementia, lived alone in her own flat within a sheltered housing building. Anthony was a friend of Mrs. Archer and her caregiver. Mrs. Archer’s kitchen was fitted with an automatic oven shut-off device to prevent potential kitchen fires (see map in Fig. 3). During the fourth visit, Anthony recounted to the fieldworker the incident. The evening before the visit, one of Mrs. Archer’s granddaughters spent the night with her. The granddaughter woke up in the middle of the night to cook food by herself then she accidentally fell back asleep only awaking to the smell of burning food. The granddaughter removed the pot from the oven and placed it on the worktop, then went back to bed. Unbeknownst to Mrs. Archer and her granddaughter, the hot pot began to melt the worktop and the cupboards above caught fire. If Mrs. Archer’s granddaughter had left the pot on the oven, then the automatic oven shut-off device may have prevented the kitchen fire.

An hour into this visit, the housing manager came to meet with Anthony to discuss removing Mrs. Archer’s oven and oven in light of the kitchen fire. She would have meals prepared by visiting care workers instead. After the housing manager left, Anthony tried to explain the decision to Mrs. Archer. At first, she did not understand, repeating her wish to continue to cook her own food, but Anthony continued to explain that it was “not safe” for her to keep her cooker. She would not able to cook her own Caribbean food anymore. She wondered aloud whether the care worker would “cook her food right”.

This case illustrates how when people involved do not understand how devices function and the processes needed to use them, then the devices cannot ‘intervene’ as intended. The consequences for such actions here included the Mrs. Archer’s kitchen eventually being abandoned except for when a care worker re-heated previously-prepared food. Mrs. Archer lost the choice to prepare her own meals due to concerns about her safety despite her not having been involved in the kitchen fire. How people use and understand ATT can directly impact their involvement in everyday activities which may have great personal significance for their identity and wellbeing.

## Discussion

The introduction of ATT illustrates how care practices could evolve from co-located, face to face interactions to be replaced with technological mediation through apps, screens, or, in some cases, displaced and disrupted outside of everyday spaces and practices. The findings suggest, however, that how many policy makers and ATT manufacturers imagine community care through ATT service provision may not reflect actual practices in technology-enabled dementia care. As previous research has also suggested, older people (with and without a dementia) and family caregivers may, contingently rather than systematically, make ATT work for them [23, 26]. ACCOMMODATE, however, highlights not only the role of caregivers to fit these technologies into care practices but also how their use of ATT can change the spaces and placement of care and everyday life [20, 53]. It shows how people’s practices with ATT shift dependencies in care arrangements [54]. These findings illustrate the limitations for ATT to enable people with dementia to ‘live independently in the community’ without support from caregivers to help adapt technologies to ‘fit’ into their lives.

Ethnographic findings illustrate how ATT provision could give caregivers an episodic sense of security or ‘peace of mind’ yet it may also introduce novel challenges for how caregivers monitor and support people living with dementia. Recognising such challenges suggests there is a need to attend to and understand the specifics of how people with dementia and caregivers incorporated ATT devices into their own spaces and routines. Previous research with older adults and people with dementia have drawn on the concept of bricolage, where people use available materials to create or adapt a new product, to explain this process of adaptation [22, 25, 26]. ACCOMMODATE findings build on this body of work to illustrate and contextualise *how* people with dementia and caregivers adapted ATT through the study’s longitudinal, observational dataset as opposed to cross-sectional interview data. Appreciating the specific context is important to appreciate fully how ATT does work as an intervention, or not. ‘Effectiveness’ of the technology was conditioned by participants’ social relations around sharing and interpreting instructions on how to use the device, and adapt to the environment, both in-home object placement and the need and behaviours of other occupants. The findings emphasise that investigating the effectiveness of ATT should involve combined and complementary studies and methods. Qualitative approaches, such as ethnography, can address how and why people with dementia and caregivers attempt to work to accommodate these devices within their everyday lives and care arrangements through focused observations and conversations about people’s practices – the ways people actively used these technologies – in their everyday lives. Technology occupies space on a person or in their home. It requires people to make choices relating to whether and how it can fit on bodies and in domestic spaces. As seen here, these choices come linked with value judgements about what care practices and interventions people find suitable in their everyday routines for people living with dementia in the community. People with dementia and caregivers appeared to first fit technologies within how they wished to live their lives. If they could not do so, then they abandoned the use of the ATT.

The diversity of ways of living with dementia, caring for a person with dementia, and accessing a wide range of differing ATT available complicates attempts to make ATT an appropriate and effective intervention for community-based dementia care. These situated cases highlight the essential mediating role played by caregivers in these processes. Individual caregivers and people with dementia may, through active negotiation and tinkering come to find a specific ATT product can then help them to manage their care responsibilities or activities of daily living in terms of their particular situation. It is important, however, that any solution that uses ATT will be time-bound, as care needs can rapidly fluctuate as a person experiences emerging limitations as their dementia progresses and fresh challenges are raised for caregivers to negotiate. Caregivers are subject to significant pressures and their capacity may change both with age and their own health. ATT provision in dementia will, therefore, require revisiting through a regular review process to accommodate such changes in the lives of the person with dementia and their caregiver. Future research should extend the longitudinal aspect of this research to explore how the disease progression of a person with dementia affects their practices with ATT.

Through such insights, we hope to illustrate precisely how research on the implementation and uptake of ATT in dementia and gerontology research can move beyond barriers and facilitators to a more a nuanced understanding in the contexts of ageing with a dementia and caregiving practices [55].

## Conclusions

These ethnographic findings flag up unintended and unanticipated consequences for ATT implementation and utilisation within ‘real world’ community-based dementia care contexts. The ethnographic approach details how people’s use of ATT shifted over time. Nonetheless, even temporary use of ATT may have deferred more complex and more acute care crises for the person with dementia or caregiver. Transient effects or limited engagement with technology should not necessarily be interpreted therefore as a failure in its uptake or effect. It underlines the need to *identify and map the context of ATT provision over time within the changing lives of people with dementia and their caregivers*, relative to service provider organisations, as these cases revealed.

This study illustrated the need to appreciate more fully the importance of people’s everyday activities and relationships in continuously shaping the context, experience and delivery of dementia care. Only through an improved understanding of practices, the array of human activities that shape and define the extraordinary and mundane aspects of everyday life with dementia, can future implementation and uptake of technology improve the effectiveness and sustainability of dementia care.

## Data Availability

The datasets used and analysed during the current study are available from the corresponding author on reasonable request.

## Abbreviations

ACCOMMODATE: A Collaborative, COMMunity-based ethnography of people with Dementia and their caregivers using Assistive technology and Telecare in England. The name of the study reported in this article
ATT: Assistive technology and telecare
ATTILA: Assistive Technology and Telecare to maintain Independent Living At home for people with dementia. The pragmatic, randomised controlled trial investigating the efficacy and cost effectiveness of assistive technology and telecare in community-based dementia care
